# Supporting the capacities and knowledge of smallholder farmers in Kenya for sustainable agricultural futures: a Citizen Science pilot project

**DOI:** 10.14324/111.444/ucloe.000065

**Published:** 2023-12-01

**Authors:** Matthew Davies, Muki Haklay, Timothy Kiprutto, Megan Laws, Jerome Lewis, Samuel Lunn-Rockliffe, Jaqueline McGlade, Marcos Moreu, Andrew Yano, Wilson Kipkore

**Affiliations:** 1 McDonald Institute for Archaeological Research, University of Cambridge, Cambridge, UK; 2 Department of Geography, University College London, London, UK; 3 Prosperity Co-Lab Africa and British Institute in Eastern Africa, Nairobi, Kenya; 4 Department of Anthropology, London School of Economics, London, UK; 5 Department of Anthropology, University College London, London, UK; 6 Institute for Global Prosperity, University College London, London, UK; 7 Prosperity Co-Lab Africa, Nairobi, Kenya; 8 School of Natural Resource Management, Department of Forestry and Wood Science, University of Eldoret, Eldoret, Kenya

**Keywords:** Citizen Science, *Sapelli*, smartphone, co-design, trans-disciplinary, farmer, agriculture, sustainability, Kenya, Africa

## Abstract

Sub-Saharan Africa is often presented as the continent most vulnerable to climatic change with major repercussions for food systems. Coupled with high rates of population growth, continued food insecurity and malnutrition, thus the need to enhance food production across the continent is seen as a major global imperative. We argue here, however, that current models of agricultural development in Eastern Africa frequently marginalise critical smallholder knowledge from the process of future agricultural design due to a lack of a methodological tools for engagement. This paper addresses this by outlining a potential means to capture and share locally produced agronomic information on a large scale. We report on a ‘Citizen Science’ pilot study that worked with smallholder farmers in Elgeyo-Marakwet County, Western Kenya, to co-design a mobile application using the well-developed *Sapelli* platform that easily allows farmers to identify, record and geolocate cropping patterns and challenges at multiple stages in the agricultural calendar using their own understanding. The pilot project demonstrated the technical and epistemological benefits of co-design, the abilities of smallholder farmers to co-design and use smartphone applications, and the potential for such technology to produce and share valuable agricultural and ecological knowledge in real time. Proof-of-concept data illustrates opportunities to spatially and temporally track and respond to challenges related to climate, crop disease and pests. Such work expounds how smallholder farmers are a source of largely untapped ecological and agronomic expert knowledge that can, and should, be harnessed to address issues of future agricultural resilience and food system sustainability.

## Introduction

Sub-Saharan Africa is often presented as the continent most vulnerable to climatic change with major repercussions for agriculture and food systems [1–3]. Coupled with high projected rates of population growth, urbanism, climate change and existing major instances of food insecurity and malnutrition, the need to enhance and secure food production across the continent is thus seen as a major global imperative. Contrasting approaches to this challenge tend to emphasise either externally driven technological innovation and ‘Green Revolution’ policies [4–6] or prioritise agroecology principles and practices and food sovereignty movements [7–9]. However, Green Revolution frameworks have been critiqued for promoting unsustainable industrial production processes and chemical inputs whilst simultaneously ignoring the unjust politics of food production and distribution [10–13]. Conversely, agroecological approaches are subject to criticism for being highly labour intensive and lacking scalability on a level that can meet Africa’s growing food demands [14,15]. More nuanced work emphasises the importance of co-production with farmers, viewing them as potentially pivotal actors within innovation systems [16–19]. Yet, whilst there is much potential for harnessing farmers innovative capacities, there are few methodologies to support such an approach and there remains a dearth of detailed information of the sophistication and complexity of farmers’ knowledge and experimental activities.

This paper addresses this lack of methodology and detailed knowledge by outlining a potential means to capture and share locally produced agronomic information on a large scale and to provide opportunities for empowering farmers to collect, share, own and act on their own data. In what follows, we report on a ‘Citizen Science’ pilot study that worked with smallholder farmers in Elgeyo-Marakwet County (EMC), Western Kenya, to co-design a mobile application using the well-developed *Sapelli* platform that easily allows farmers to identify, record and geolocate cropping patterns and challenges at multiple stages in the agricultural calendar. The pilot project demonstrated the technical and epistemological benefits of co-designing such applications, the abilities of smallholder farmers to co-design and use smartphone applications, and the potential for such technology to enable the production and sharing of valuable agricultural and ecological knowledge in real time. Such work seeks to expound how smallholder farmers are a source of largely untapped ecological and agronomic expert knowledge that can, and should, be harnessed to address issues of future agricultural resilience and food system sustainability.

## Background

Smallholder farmers in sub-Saharan Africa are commonly presented as relatively static, resistant to change and lacking the capacities to innovate in the face of population growth, food insecurity and climate extremes. Such stereotypes fuel calls for external interventions that can all too easily by-pass existing sustainable farmer knowledge and practice and, in the process, fail to understand how external innovations are not simply adopted passively by recipient farmers, but rather creatively incorporated into existing bodies of knowledge and practice [20]. Consequently, many agricultural interventions have either fostered unwanted outcomes or outright failed in their attempts to increase livelihood wellbeing and build agronomic resilience [17,21,22].

We propose that a fundamentally different approach is needed that places farmers at the centre of design processes in order to harness local knowledge and practice and collaboratively build sustainable socially and culturally appropriate agricultural futures [23]. In doing so, we advocate for a framework that aligns itself not only with calls for agricultural production rooted in localised ‘food sovereignty’ movements [9,12], but with research trends that allow farmers to take back a central role in the design, experimentation and validation process of agronomic knowledge creation [24,25].

The foundations for this argument build from an extensive but poorly understood body of literature that has explored the deeper histories and ethnographies of the long-term sustainability of intensive and highly productive agricultural practice across many regions of Eastern and Southern Africa [26–30]. This body of work, alongside more recent research on farming innovation within the research region,^1^ collectively illustrates how farmers in the study region continually engage in creative processes of innovation, selective valuation and intelligent adoption of old and new technologies on a daily, weekly, monthly and seasonal basis. This innovation underpins a spatially and temporally variable diversity of cropping and agronomic practices that we have outlined elsewhere and which demonstrate the value of farmer knowledge to food security and climate resilience. The pilot project outlined here thus offers the potential to extend empirical analysis of such complexity and enhance the evidence base in support of farmer-led resilience.

Importantly for this project, smallholder farmers in the study region and across Eastern Africa are technologically savvy. In Kenya, for example, the term ‘digital farmers’ constitutes a Facebook group with over 400,000 active members engaging in dynamic forms of information sharing and knowledge exchange. Digital engagement is also evident throughout a plethora of smartphone applications already targeted at African farmers, such as iCow and WeFarm. Whilst no doubt useful tools for many farmers, such applications and digital platforms are normally externally designed technologies that offer farmers products or services (finance, marketplaces) that tie them into certain kinds of market relations in ways that can undervalue the abilities, knowledge and networks of farmers themselves.

As we explore below, we advocate for alternative approaches that build upon farmers’ knowledge of smartphone and mobile Internet technologies in ways that more readily facilitate the co-design of tools that enhance their own agricultural capacities. At the same time, this project was embedded in longer-term research that seeks to understand the history, contemporary practice and potential of farmer-led agricultural innovation in Elgeyo-Marakwet, thus providing an effective context in which to undertake such pilot research.

## Harnessing smallholder farmer’s capacities: a Citizen Science approach

Citizen Science has multiple distinct approaches, phases and degrees of citizen engagement [31–33]. In recent years, the core of Citizen Science work has aimed to empower communities to build, design and then utilise their own research potential. As explored elsewhere [34], a majority of Citizen Science projects have focused on a highly specific problem or issue identified by outside ‘experts’. The gathering of data is then used to build an evidential base for or against a certain course of action – such as noise pollution, illegal logging, community rights to land or something similar [35,36]. Mostly, these problems are identified, and projects designed by scientists and researchers who then ask the public to join in by carrying out research tasks that can range from carrying out experiments, collecting data or analysing images. Less common are Citizen Science approaches designed to broadly capture the knowledge and practices of a community as well as their own understanding of the challenges or issues that they currently face.

In 2019, this pilot study worked with a small community of smallholder farmers in EMC, Kenya, to apply the Extreme Citizen Science approach ([37]; see https://uclexcites.blog) and trial a process of Citizen Science research co-design. Working with *Sapelli collector*, a highly customisable mobile data collection application designed to facilitate more inclusive Citizen Science, we used a series of focus groups with n=15 farmers including men and women, to explore cropping practices and challenges faced at each step in the annual cultivation cycle. Information gleaned from initial focus group discussions was then used to co-design a *Sapelli* project with the participants. A *Sapelli* project defines the user and pictorial interfaces that are displayed through the *Sapelli collector* mobile app (see below for more detail). From the original 15 farmers, a sub-group of six individuals were given a smartphone and trained to use the interface of the *Sapelli* project. With airtime and technical support for a period of four months, the farmers had the opportunity to test the data collection process and to feedback on the application design for subsequent refinement.

After the pilot period, we ran focus groups where the advantages and disadvantages of the application were discussed in detail, allowing us to adjust for future use. This review particularly focused on the changes that might be required to scale up the use of the application by farmers and to enhance its usefulness to these users. In the section that follows, we further elaborate on this co-design process and on the structure and function of the resulting *Sapelli* application. Following this, we report the results of the pilot data collected and explore the potential analyses and uses that these data might facilitate. In the final section we reflect on the pilot project to date, report on the feedback from farmers, and outline future steps for Citizen Science approaches to agricultural sustainability in Kenya with potential applications to other locations in Africa and elsewhere.

## Engagement and participatory design

The processes and principles of co-design are key to foregrounding the value of local knowledge and practice, not least because it is farmers who manage and curate the agricultural landscapes of EMC and who have lived experience of the kinds of challenges encountered and solutions needed over daily and seasonal timescales. Taking time to understand the nuances of these lived experiences has a direct impact on the kinds of otherwise unknown/unforeseen information that needs collating, including, for example, different methods for managing soil fertility or varying issues encountered across ecologies. Co-design also helps to think through a number of practical considerations and ethical implications that make for a more equitable project that is not exploitative of people’s knowledge, time or resources. For instance, participants know the limits of available resources (e.g., regional phone signal, smartphone charging, cost of airtime), may have a range of other livelihood commitments and time constraints (e.g., domestic chores, farm preparation, small scale enterprises) or may want to highlight a range of ethical concerns (e.g., capturing and storage of personal data). This process gives participant farmers a sense of intellectual and methodological ownership of the project.

**Figure 1:**
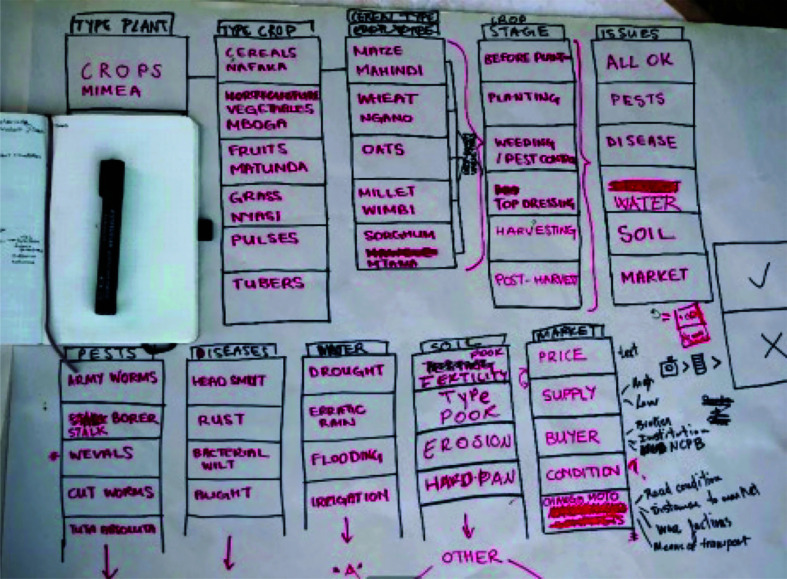
Draft of the co-created paper prototype, which is later on converted into a *Sapelli* project.

In view of this, the co-design process for the *Sapelli* project followed that outlined in Moustard et al. [34] and was built on the core principle of free, prior and informed consent (FPIC) and the development of a community protocol to govern the collaboration. This not only involved providing participants with all of the information on the proposed project, but also the active collaborative exploration between researchers and community members to understand potential positive and negative impacts of the project’s outcomes. The first phase of work focused on engaging with farmers and agricultural extension officers in order to identify what farmers want to report and map. It was here that the first paper prototype was developed during a participatory design session (Fig. 1). Following this, a further session was organised with a small group of farmers and agricultural extension officers to refine the paper prototype and create the pictograms and images that represent the crops and farming issues as identified and classified by the farmers themselves. While *Sapelli* is designed to be inclusive for people without literacy, the use of pictograms and images can be mixed with text where it is appropriate, thereby increasing accessibility for people with medium-to-higher levels of literacy. In this project the farmers chose to combine easily recognisable pictograms with Kiswahili words.

Participatory design is a process that requires multiple paper and digital iterations and real-time coding and field testing. While the process may face time and resource constraints, the well-established *Sapelli* co-design process allows a working prototype for extensive field-testing to be designed in a matter of days. Nevertheless, due to time constraints, the appropriateness of the pictorial interfaces and the navigation flow was tested and discussed with only a few farmers and improvements were based on these few farmers’ feedback and the knowledge of the extension officers. Plenary focus groups suggested that the interfaces and navigational flow worked well with only minor alterations suggested.

## The *Sapelli* project structure

**Figure 2:**
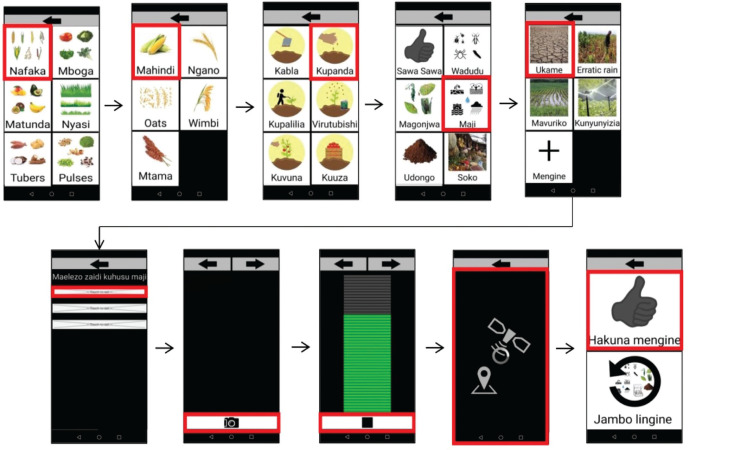
The *Sapelli* co-designed project, showing the workflow and pictograms (with captions in Kiswahili) displayed when cereals and water issues are selected, cereals> maize> water problems> drought> additional text> photograph> voice recording> geolocation> finish/another issue.

**Figure 3:**
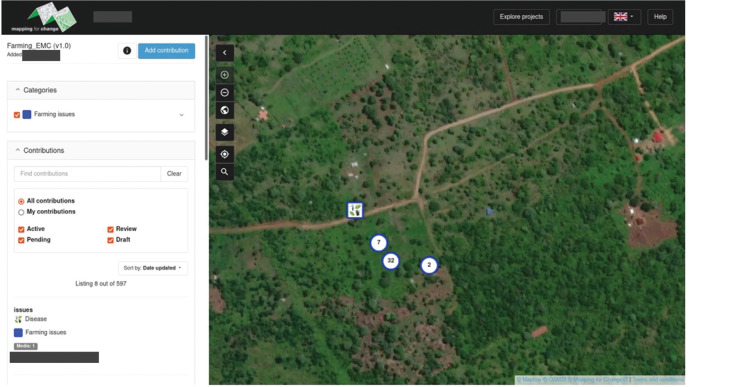
Screenshot of Community Maps showing some of the data points collected by farmers to report farming issues.

The technological output of the participatory design process is the *Sapelli* project that defines which pictograms will be used, how they are displayed in the *Sapelli collector* mobile app and what data are stored and transmitted to the database management platform GeoKey (https://geokey.org.uk/). This latter is designed to support participatory mapping and visualisation in the Community Maps’ user-friendly interface (https://communitymaps.org.uk/). The *Sapelli* project was co-designed to collect and share information about crop type, stage and farming issue. The 33 crops/plants identified were grouped into six categories (cereals, vegetables, fruits, grass, tubers and pulses) by the farmers. After the crop/plant is selected, the user is then prompted to select the stage of the cropping cycle (before planting, planting, wedding/pest control, top dressing, harvesting or post-harvesting), and then the type and specificities of any issue (pests, disease, water, soil, market or equipment) currently being faced. The next screens allow the users to provide additional information using text, audio recording or by taking a picture. When the user reaches the last screen, the location is recorded, and the user can either record another issue or store and send the data (the data is automatically sent when online and the region was well covered by 3G/4G). Figure 2 shows the workflow as co-designed and illustrating the visual interface supplemented by written terms in Kiswahili. Figure 3 shows an example of project data and location, with the attribute information of the contribution selected in the map shown in the panel on the left.

## Data collection: results and preliminary analysis

**Figure 4:**
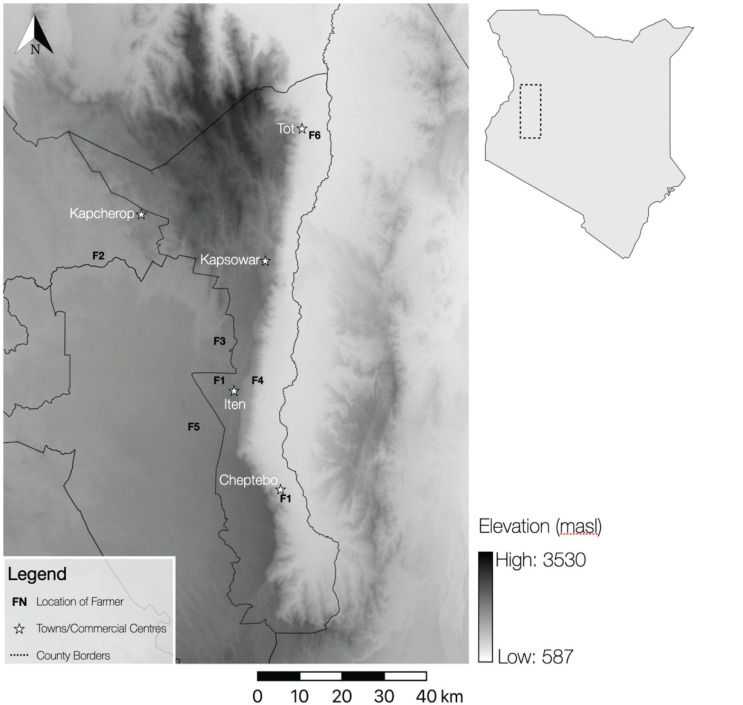
Location of Farmer Activities in and around EMC.

Data collection was undertaken by six farmers (F1–6; five male, one female) from April to August 2019 in and around EMC (Fig. 4). All data points collected by farmers were geolocated, but in order to preserve anonymity, locations of research activities are presented here at a low resolution by clustered points according to Device ID (i.e., the smartphone being used by one farmer). Spatial documentation within the context of this project is particularly interesting given that farming practices can vary greatly across the County. This variation is due to the altitudinally contingent ecological and climatic diversity of the region, where the acacia scrubland environment of the semi-arid Kerio Valley (c. 1000 masl) is on average hotter and drier than the forest environments of the highlands (c. 2500 masl).

**Figure 5:**
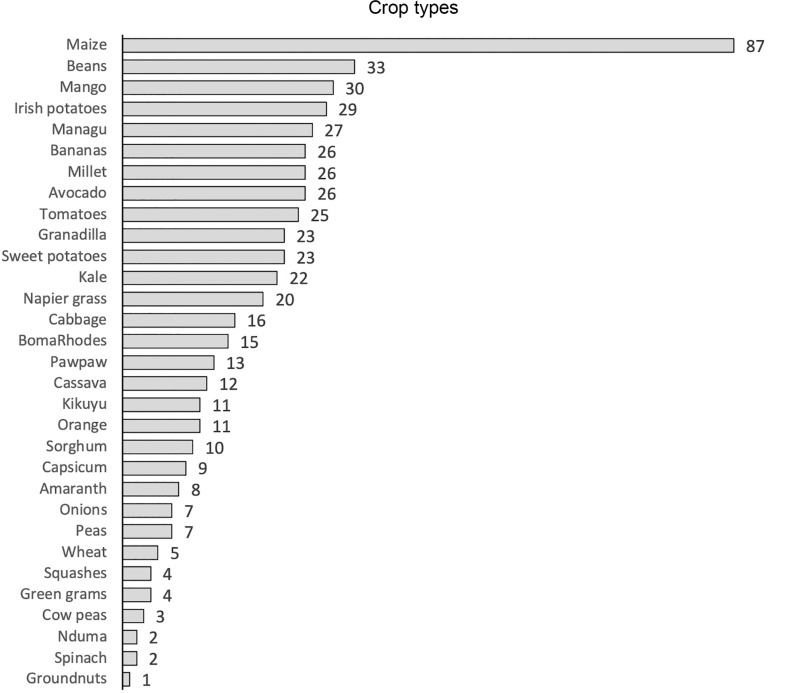
Total number of data points for different foodstuffs collected by the six farmers.

Farmers 2, 3, 4 and 5 undertook work in the highland areas between c. 2000 and 2300 masl, and Farmer 6 collected data in the Kerio Valley in Tot-Sibou village (c. 1000–1200 masl; Fig. 4). Farmer 1 recorded data from two plots of land, one in the highlands and one in the valley. A total of 534 data points were taken across the devices, with a mean average of 89 points per device. Across all locations, a total of 31 different foodstuffs were documented (Fig. 5), with each farmer recording a mean average of 17 different foodstuffs. Of the total amount of crops grown, 39.9% were exclusive to the highlands. All foodstuffs that were grown in the valley were also grown in the highlands, albeit in much smaller proportions (e.g., mango trees accounted for 21.4% of crops grown in the valley and only 0.7% of those grown in the highlands).

**Figure 6:**
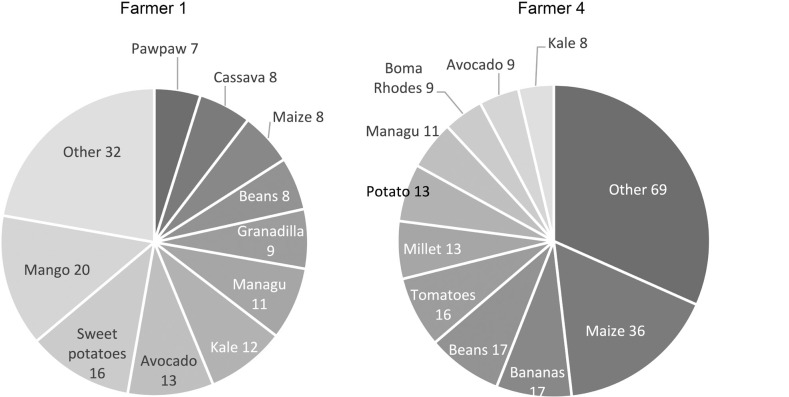
Recorded crops grown by Farmer 1 and Farmer 4 (see Fig. 4 for location).

The data captured clearly demonstrate the potential for better understanding and exploring farmers, highly resolved micro-ecological knowledge, by illustrating the differing combinations and ratios of crops grown at varying altitudes. For example, as shown in Fig. 6, Farmer 1 grows 22 different crops in combination, with the top 5 recorded being mango (20), sweet potato (16), managu (African nightshade) (11), avocado (13) and kale (4). Farmer 4 grows 28 different crops, but the top 5 recordings are very different: maize (36), tomatoes (16), potato (13), beans (17) and bananas (17). Similar diversity is found across the other farmers, including surprising diversity within ecological/altitudinal zones. This provides a powerful example of the diversity of farmer practice and farmer-led experimentation recorded in previous smaller-scale and qualitative research [20]. These data further speak to opportunities for targeted support with different crop species and the sharing of effective botanic ecological knowledge among farmers in different zones and regions. When collected and analysed longitudinally, such data would also offer the potential to understand longer-term changes to cropping patterns, markets and changing ecological and climatic conditions.

Data points for each crop also captured the stage of planting and any associated challenges with considerable potential for real-time mapping of the distribution of disease, pests and climatic events such as drought or flooding and the linking of these to critical moments in the cultivation cycle. The greatest percentage of data points were taken at the stages of Weeding/Pest Control (35%) and Top Dressing (33%), followed by Planting (11%), Harvesting (11%), Post-Market (7%) and Before Planting (3%). Issues and challenges documented suggest that 95% of recorded data points had at least one associated issue, of which 31% were related to disease (167 instances of blight, rust, bacterial wilt, head smut or unidentified pathogens). Challenges related to water, including flooding, erratic rain, drought or irrigation, accounted for 29% of the issues encountered. Problems associated with market access and price (14%) were greater than those associated with pests (9%), soil conditions (9%) or equipment (1.5%).

**Figure 7:**
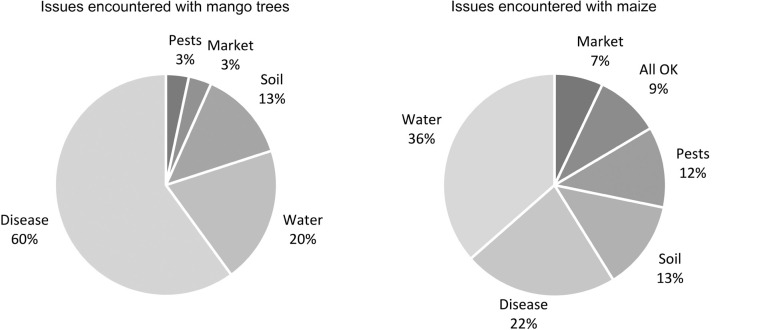
Comparison between challenges associated with mango trees and maize.

Whilst correlations between crops grown, stages of production and issues encountered remain tentative, it is possible to pick out some relationships from the pilot data. For example, certain crops present distinct challenges, with approximately 60% of mango trees displaying issues with disease in comparison to 22% of maize crops (Fig. 7).

**Figure 8:**
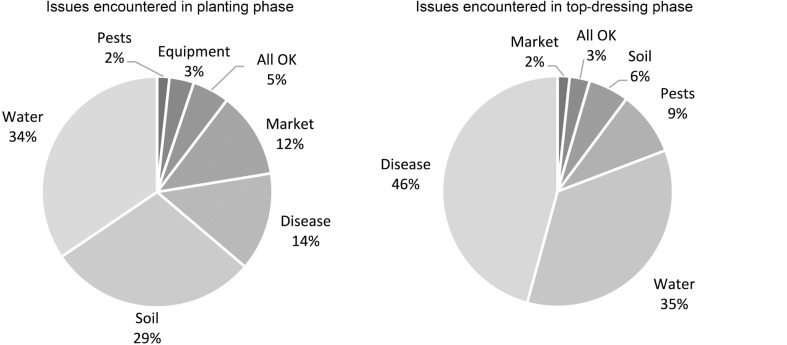
Comparison between cropping challenges at different phases of the cropping cycle.

Similarly, there are clear differences in the problems being encountered by farmers at the different phases of the cropping cycle, where the challenges associated with poor soil conditions at the planting phase (29%) are far more prevalent than at the top-dressing phase (6%), where disease is more common (Fig. 8).

**Figure 9:**
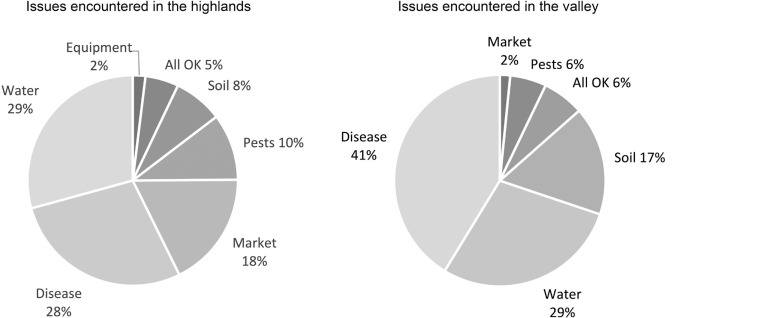
Comparison between cropping challenges encountered in the highlands vs. the Kerio Valley.

The spatial distribution of issues encountered by farmers in different locations can also be observed from the data (Fig. 9). The greatest issue encountered at lower elevations was disease, accounting for 41.3% of the challenges of valley-grown foodstuffs, followed by water (28.6%) and soil (16.7%). The greatest challenges in the highlands were water-related (29.3%), followed by disease (28%) and market access (17.8%). As discussed later, such data clearly lend themselves towards spatially targeted interventions and support.

These preliminary results demonstrate that farmers are planting a huge diversity of crops in interesting and complex ways and have a clear understanding of the problems related to the health of farm produce and market infrastructures. Before we discuss the implications of these data, it is important to point out that preliminary correlations from this analysis between location, crop types and issues encountered must be treated with caution. Indeed, these are not the results of a replicable large-scale technical study with strict controls for measuring specific variables. Rather, data should be seen as proof of concept for harnessing farmer-led data collection procedures and producing an emergent dataset that captures the ways in which smallholders observe and address multiple issues across time and space.

## Discussion

The results from this pilot study demonstrate the vast potential behind citizen-led data collection. In the first instance, it allows for the large-scale mapping of farming innovation and experimentation in response to multiple challenges and issues being encountered on a daily, monthly and seasonal basis. With this kind of information being gathered via *Sapelli collector*, real-time data may be shared, and knowledge exchanged between farmers, extension officers and other practitioners within wider agricultural value chains. Participant farmer feedback suggested that the integration of such technologies into messaging (text, images, audio and maps) services for both farmer-to-farmer and extension officer knowledge transfer can help to provide live updates to the challenges and solutions of farmers from across the region (and beyond). If integrated into novel knowledge networks (messaging, open access forums), such technologies may both support immediate livelihoods and stimulate new farmer-led innovations. User focus group feedback sessions emphasised the need for better knowledge sharing across the project, including the ability to see data and information collected by others and the ability to allow farmers to share specific knowledge about cropping challenges by, for example, commenting directly on the data collected by others. Feedback also noted the need for better provision for access to airtime and data perhaps through an automated system using MPesa. Finally the pilot users noted some challenges with slow data syncing and the need for a refined interface for managing data synchronisation. These challenges can be addressed by the technical team in future iterations. Nevertheless, feedback was highly positive as to the effectiveness and ease of use of the interface and the potential of the project for knowledge sharing and advocacy.

More urgently, equipping farmers with the ability to upload real-time challenges may help to capture the changes and impacts associated with climatic events and the movement of disease and pests at multiple spatial (regional, national, international) and temporal scales (hours, days, weeks, months). A concrete example of this can be seen with the early warning systems for monitoring locust swarms that are available on the Locust Hub (https://locust-hub-hqfao.hub.arcgis.com). The data for this is collected on the ground by trained field staff using specialist software and hardware, notably the Food and Agriculture Organization (FAO) developed eLocust3 monitoring system that offers near-real time data validation and tracking of locust swarms (https://www.fao.org/fao-stories/article/en/c/1244192/). Whilst perhaps effective for planning and implementing control operations, using eLocust3’s technical software and hardware requires multiple training and refresher sessions. As such, the scale at which locust swarm monitoring can be implemented and shared is compromised, thus reducing the spatiotemporal resolution of locust activity that may otherwise be invaluable for the preparedness of everyday farmers.

By contrast, the existence of pests such as locusts was easily coded into the *Sapelli* project and would allow farmers to record their presence as part of wider data gathering on agricultural practice and challenges and using a simple smartphone interface rather than specialist equipment. With enough participation, real-time farmer-generated data on desert locust behaviour and movement may be able to provide more fine-grained live information on the time, location and direction of movement of locust swarms or hopper bands. This concept need not only be applied to desert locust swarms, but also the monitoring of pest or disease breakouts such as fall armyworm or blight, as well as the sharing of information on how farmers innovate and experiment to build on-farm resilience and prepare for future challenges.^2^

The broader point here is that existing systems that monitor ecological and socioeconomic change may lack the ease of use, number of users and granularity of data needed for meaningful local and regional policy making both over the long-term and in emergency or crisis situations. Such policies are commonly based on syntheses that make poorly articulated assumptions about local effects, impacts and mitigation. At best these fail to harness the knowledge potential of farmers and at worst can stand at odds with the perceptions, experiences and aspirations of the communities they aim to assist. With the advent of affordable smartphones and mobile broadband (4G and 5G) across Eastern Africa, it is now possible to work closely with rural communities to collect information on a wide number of ecological and social issues, even where numeracy/literacy levels are low. Working with policy makers to build farmer-led live information systems may prove incredibly important for enhancing and empowering farmer knowledge and circulating this knowledge to increase preparedness for multiple challenges.

## Conclusion

The indigenous African plant knowledge and sustainability project demonstrates the significant potential behind the co-design of Citizen Science data collection. Whilst the results from the pilot work presented are tentative, the value of this project has been to provide a novel example of how processes of co-design and principles of collaboration are integral for foregrounding local knowledge and practice. The genuine engagement of farmers and extension offices throughout the process helps to not only create an accessible and appropriate user interface for a mobile technology, but also starts with farmer priorities, wants and needs rather than imposing a research agenda upon them. We see this as a necessary step in empowering farmers to design their own futures [23] and towards advocating for better informed policy making and crisis management. Whilst still in its infancy, we envisage these methods of co-design and data collection will continue to be refined and scaled (see https://uclexcites.blog), and for appropriate technical upgrades to be implemented to enhance the knowledge sharing potential of this and similar *Sapelli* projects.

## Data Availability

The datasets generated during and/or analysed during the current study are available from the corresponding author on reasonable request. *Sapelli* collector is fully open source and can be downloaded freely and used via the Google Play Store or GitHub repository. *Sapelli* packager is available via GitHub. The proof-of-concept data collected under this project includes personal spatial data and is currently not publicly available.

## Notes

1 See https://seriouslydifferent.org/igp-stories/reconceptualising-innovation-for-agricultural-futures-in-africa-part-i and https://seriouslydifferent.org/igp-stories/reconceptualising-innovation-for-agricultural-futures-in-africa-part-ii.

2 Alternatively, separate *Sapelli* projects for various forms of pest monitoring might easily be co-designed.
